# Opposite effects of estradiol and progesterone on woman's disgust processing

**DOI:** 10.3389/fpsyt.2023.1161488

**Published:** 2023-04-05

**Authors:** Mei Liu, Xia Zhang, Zhengming He, Yuan Liang, Bihong Zou, Xianjun Ma, Simeng Gu, Fushun Wang

**Affiliations:** ^1^Institute of Brain and Psychological Science, Sichuan Normal University, Chengdu, China; ^2^Department of Neurology, Affiliated Lianyungang Hospital of Chinese Medicine, Nanjing University of Chinese Medicine, Nanjing, China; ^3^Department of Psychology, Jiangsu University Medical School, Zhenjiang, China

**Keywords:** ovarian hormones, menstrual cycle, disgust, SC-IAT, rs-fMRI

## Abstract

**Background:**

Ovarian hormones play a critical role in emotion processing, which may be a major reason for the high rates of major depressive disorders in women. However, the exact roles of estradiol and progesterone in emotional processing remain unclear. To this end, we performed behavioral and rs-fMRI studies on the effects ovarian hormones on disgust emotion.

**Methods:**

In Experiment 1, 95 Chinese female undergraduates completed the single category implicit association test (SC-IAT) and explicit measures of disgust intensity task, 32 in the menstrual phase, 30 in the follicular phase, and 33 in the luteal phase. In Experiment 2, A total of 25 healthy female undergraduates completed three sessions of the rs-fMRI. The menstrual group was scanned during cycle days 2–5, the follicular group during cycle during days 10–13, and the luteal group was scanned 3–7 days before the next menstruation.

**Results:**

The behavioral results showed that women during the luteal phase had higher D scores and shorter response times (RTs) to disgust stimuli compared to the menses and follicular phases. In contrast, women during the follicular phase had fewer feelings of disgust and longer RTs to pathogen stimuli compared with that during the menses and luteal phases, but this effect was moderated by the intensity of the stimuli. rs-fMRI studies showed that women during the luteal phase have higher functional connectivity in the salience network than those in the follicular phase. Compared with the menstrual phase, women have lower functional connectivity in the amygdala during the follicular phase.

**Conclusion:**

In summary, a more negative attitude to disgust stimuli and the enhanced functional connectivity of the salience network during the luteal phase may be associated with high progesterone levels, whereas lower disgust feelings and reduced functional connectivity of the amygdala during the follicular phase may be associated with high estradiol levels.

## 1. Introduction

Disgust is one of the least studied basic emotions, but it has recently been found to be the major cause of most affective diseases (1, 2) such as major depressive disorders (2, 3), anxiety disorders (4, 5), and eating disorders (6, 7). In our daily lives, common elicitors such as pathogenic cues (e.g., bacteria, viruses, and parasites) or moral violations (e.g., incest, theft, and violence) induce disgusting feelings and avoidance behaviors (8–10). The former class of disgust is referred to as pathogen disgust, whereas the latter is known as moral disgust. Recent studies have shown that women are characterized by higher disgust sensitivity, avoidance behaviors, and physiological reactions than men (11–13). This immunological advantage may be related to certain hormones in women (e.g., estrogen, progesterone, and oxytocin) that cause them to be more sensitive to disease cues, especially during pregnancy and certain phases of the menstrual cycle (14, 15). However, this cyclical experience (fluctuating hormonal changes) may also affect the mental health of women. For example, one study found that almost 50–80% of women of reproductive age reported mild premenstrual syndrome (PMS) symptoms such as irritability, anxiety, and depression, as well as physical symptoms such as bloating and breast tenderness (16). These, in turn, impair women's daily work, social activities, and interpersonal relationships (17). Therefore, it is necessary to focus on women's hormonal status when exploring emotional processing.

In previous studies, the menstrual cycle has usually been divided into the menses or early follicular phase (low estrogen and low progesterone), follicular or late follicular phase (high estrogen and low progesterone), and luteal or mid-luteal phases (high estrogen and high progesterone) (18, 19). Therefore, the effect of hormones on emotion processing can be explored by comparing different cycle phases; however, the findings have yielded mixed results. According to the compensatory prophylaxis hypothesis (CPH), progesterone in the luteal phase increases feelings of disgust and activates the behavioral immune system (20, 21). Several studies have provided evidence of this. For example, Fleischman et al. (20) and Zelazniewicz et al. (22) showed a positive correlation between progesterone and the feeling of disgust to pathogenic cues only in the luteal phase. Pilarczyk et al. (23) found that women spent less time gazing at pathogenic pictures and had more feelings of disgust and avoidant behaviors during the luteal phase than during the early follicular phase. However, some longitudinal studies did not find that pathogen disgust or moral disgust was upregulated in the luteal phase or tracked changes in women's hormone levels (24, 25). The effect of estrogen on disgust processing has been less studied than that of progesterone. Most studies found no correlation between estradiol levels and pathogen disgust or between estradiol levels and moral disgust at different cycle phases through direct self-reports (21, 22, 24). Only a few studies have focused on the effects of estrogen on facial expression processing and demonstrated an inhibitory effect of estradiol on disgust expression recognition (26, 27). Therefore, whether pathogen and moral disgust are influenced by estrogen and progesterone needs to be further explored.

Furthermore, while estradiol and progesterone receptors are present in several brain regions that are critical for emotion, such as the amygdala and salience networks (SNs), little is known about the brain mechanisms that mediate hormone-related negative emotions (28–30). High levels of ovarian hormones, particularly progesterone, produce augmented amygdala responsiveness to negative stimuli, representing an increase in stress reactivity similar to that observed in affective disorders and in healthy women during the luteal phase (31, 32). Similarly, higher activity and connectivity in SNs have been consistently reported during the luteal cycle phase and shown to be related to higher progesterone levels both at rest and during tasks (31, 33). However, in contrast to the effects of progesterone, estradiol seems to be more associated with positive effects in terms of both mood and cognition (34). Rising estradiol levels in the late follicular period are associated with a decreased response in the amygdala and other affective regions (e.g., the hippocampus, orbitofrontal cortex, and anterior cingulate gyrus) relative to the low-hormone early follicular phases (35–37).

Although previous results have varied somewhat across studies, this is probably due to the use of self-report measures, such as scales or single ratings, which may be influenced by self-consciousness, especially for moral stimuli. Another potential methodological aspect is that a few studies used disgusting pictures or videos (21) but indicated inconsistent results, probably owing to the ceiling effect of stimuli inducing too high a disgust intensity, which results in the effects of estradiol and progesterone being masked. In addition, sex hormone-modulated changes in functional brain organization have been discovered in task brain imaging data for emotion-related changes (19, 38). However, the literature on how resting-state networks are affected by ovarian hormones is limited.

The single category implicit association test (SC-IAT) (39) is a variation of the IAT devised to capture evaluations of the object, such as different types of disgust phrases. Compared with self-reports, the SC-IAT is relatively unsusceptible to faking or self-presentational concerns. Hence, we used SC-IAT in Experiment 1 to determine the specific influences of the two ovarian hormones on the pathogen and moral disgust processing across the menstrual cycle. In Experiment 2, we further explored the effects of the menstrual cycle on intrinsic connectivity networks related to emotion processing (the amygdala and SNs). Specifically, we predict that (1) women in the luteal phase (higher progesterone levels) will have more negative attitudes and feelings toward disgust stimuli and enhanced resting-state functional connectivity in the amygdala and SNs and (2) women in the follicular phase (higher estradiol levels) will have lower disgusting feelings and reduced resting-state functional connectivity in the amygdala and SNs.

## 2. Methods

### 2.1. Experiment 1

#### 2.1.1. Participants

A total of 95 Chinese female undergraduates were recruited from Sichuan Normal University in Chengdu: 32 in the menstrual phase (2–5 days after the start of menstruation), 30 in the follicular phase (10–13 days after the start of menstruation), and 33 in the luteal phase (3–7 days before the next menstruation) were enrolled in the experiment. Subjects were screened based on the following criteria: regular menstrual cycle (24–35 days, with each period no more than 5 days early or late) for 3 consecutive months, with a menstrual flow of 4–7 days; no contraceptive or other hormonal drugs in the last 6 months; no pregnancy or breastfeeding in the last 6 months; ability to report the start date of the last three menstrual cycles; and right-handed, with normal or corrected normal vision. At the 1-month post-experimental follow-up, seven subjects experienced early or delayed menstruation and were excluded from the follow-up analysis. Thus, only 88 subjects were included in the analysis: 29 in the menses phase, 29 in the follicular phase, and 30 in the luteal phase, aged 17–25 years, with a mean age of 19.75±1.38 years. The participants' age did not differ among the three groups, *F*_(2, 85)_ = 0.047, *p* = 0.954. All participants provided written informed consent before participating in the study. The study was conducted in accordance with the Declaration of Helsinki and reviewed by the local ethics committee of Sichuan Normal University.

#### 2.1.2. Materials

The disgust and neutral phrases were carefully selected in two separate pilot studies. First, using the self-assessment manikin (SAM) method, 30 participants were asked to assess the degree of moral violation (weak to very strong), arousal (very calm to very exciting), valence (very unpleasant to very pleasant), intensities (weak to very strong), and familiarity (very unfamiliar to very familiar) of 220 Chinese phrases on a 9-point Likert-type scale. They were then placed into different emotional categories: happy, fearful, sad, disgusted, angry, and neutral. In total, 30 pathogen disgust phrases (e.g., touch feces), 30 moral disgust phrases (e.g., drug abuse), and 30 neutral phrases (e.g., phone calls) were selected according to the methods of Moll et al. (40) and Scott et al. (41) (for more details, see Supplementary material).

#### 2.1.3. Experimental procedures

Testing sessions were conducted only in the afternoon between 2:00 p.m. and 6:30 p.m. One day before each visit, the participants were instructed to abstain from consuming alcohol and caffeine, brushing their teeth, and smoking within 3 h of the beginning of the experiment. Furthermore, they were requested not to eat a meal or consume dairy products within 1 h of the experiment. Before the formal experiment, each participant provided written informed consent. On the day of the visit, participants completed questionnaires and two behavioral tasks.

##### 2.1.3.1. Single category implicit association test (SC-IAT)

We used a modified version of the SC-IAT (39). The evaluative dimension was labeled good and bad, and the object was the disgusting phrase. Thus, participants were required to complete two SC-IAT tasks (pathogen disgust and moral disgust), and the order of the two tasks was counterbalanced. Each SC-IAT included two stages, each of which consisted of 24 practice trials immediately followed by 48 formal test trials. The first stage was a compatible task in which disgust phrases and bad words (e.g., pain, illness, and evil) were categorized on the K key, and good words (e.g., goodness, loveliness, and beauty) were categorized on the S key (see Table 1).

**Table 1:** SC-IAT.

**Block**	**No. of trials**	**Left key response (K)**	**Right key response (S)**
Compatible practice	24	Disgust phrase—bad words	Good words
Compatible test	48	Disgust phrase—bad words	Good words
Incompatible practice	24	Bad words	Disgust phrase—good words
Incompatible test	48	Bad words	Disgust phrase—good words

In an attempt to prevent response bias from developing, disgusting phrases, bad words, and good words were not presented at equal frequency but rather at a 7:7:10 ratio so that 58.4% of correct responses were on the K key and 41.6% of correct responses were on the S key. The second stage was an incompatible task in which bad words were categorized on the K key and disgust phrases, and good words were categorized on the S key. Disgust phrases, bad words, and good words were presented at a 7:10:7 ratio so that 58.4% and 41.6% of correct responses were on the S and the K key, respectively. The order of the compatible and incompatible tasks was counterbalanced.

**Figure 1:**
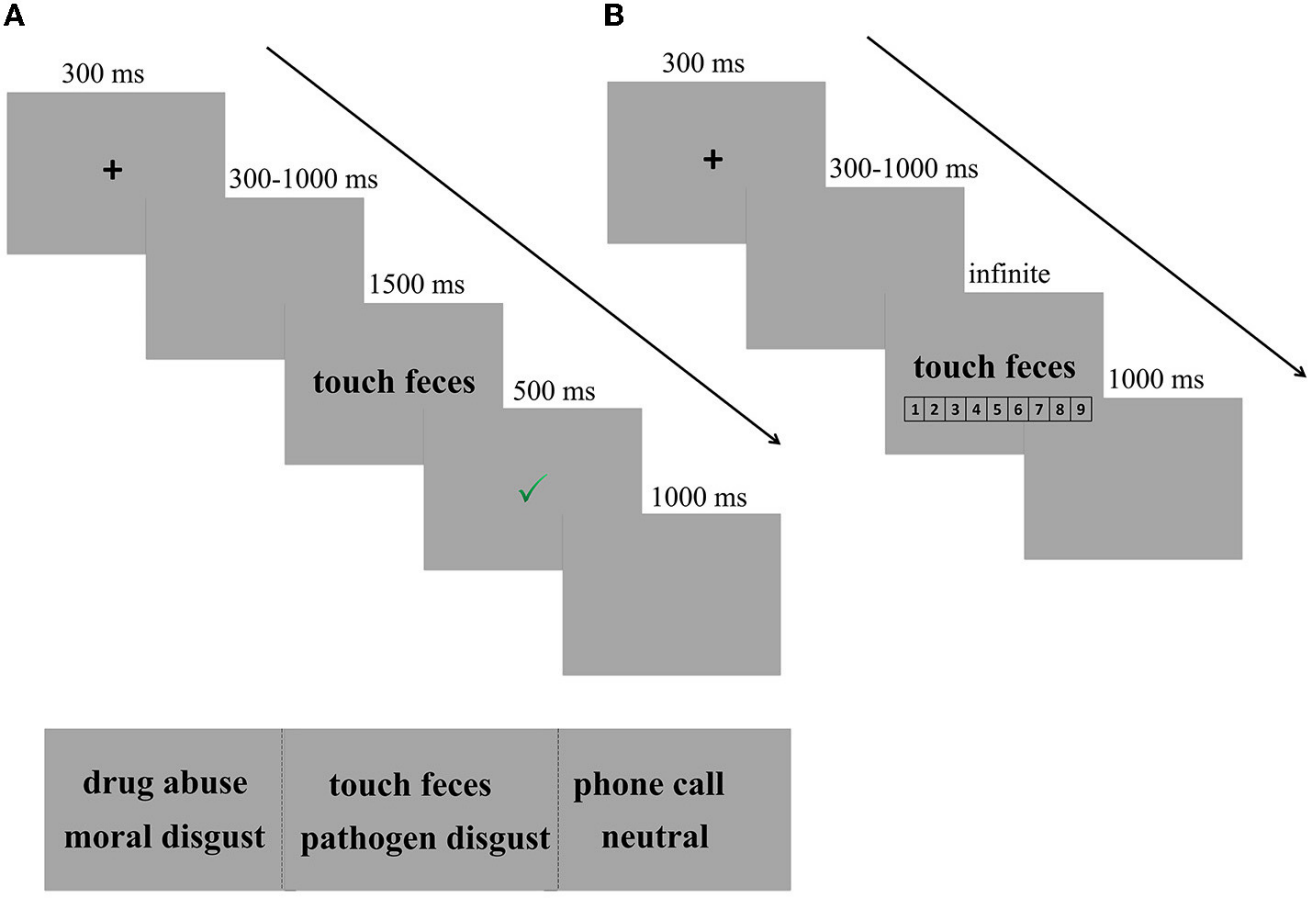
Experimental design. **(A)** Trial of example in SC-IAT. In each trial, a fixation cross was presented for 300 ms, followed by a blank screen ranging from 300 to 1,000 ms. Then, the disgusting phrase (e.g., touch feces) remained on the screen until the participants responded or for 1,500 ms, followed by a feedback for 500 ms. Finally, there was a fixed blank for 1,000 ms, indicating that one trial was completed. **(B)** Trial of example in explicit measures of disgust intensity. After completing the SC-IAT, participants were required to rate the intensity of the disgusted feelings provoked by each phrase on a scale from 1 (not disgusting at all) to 9 (very disgusting).

In each trial, a fixation cross was presented for 300 ms, followed by a blank screen ranging from 300 to 1000 ms. Then, the disgusting phrase (e.g., touch feces) remained on the screen until the participants responded or for 1,500 ms. Following each response, the participants were given feedback regarding the accuracy of their responses. A green “✓” and a red “ × ” in the center of the screen followed correct and incorrect responses, respectively, for 500 ms. However, if participants failed to respond within 1,500 ms, a reminder to “Please respond more quickly!” appeared for 500 ms. The inter-trial interval was 1,000 ms (see Figure 1A for the experimental design).

##### 2.1.3.2. Explicit measures

After completing the SC-IAT, participants were required to rate the intensity of the disgusted feelings provoked by each phrase on a scale from 1 (not disgusting at all) to 9 (very disgusting). Participants were first presented with a practice block of five trials to familiarize themselves with the task and then the test procedure, which had only three blocks. Each block had 30 trials (10 pathogen disgust phrases, 10 moral disgust phrases, and 10 neutral phrases). After each block, participants had a short break of 1 min. Every trial started with a central black fixation for 300 ms, followed by a blank screen with a jittered duration ranging from 300 to 1,000 ms. Then, the target phrase (e.g., touch feces/drug abuse/phone call) was presented in the center of the screen until the participants responded. The trial ended with a blank screen with a duration of 1,000 ms. Three types of phrases were presented in a random order (see Figure 1B for the experimental design).

#### 2.1.4. Data analysis

SC-IAT scores were computed using the newer *D*-score algorithm, referring to the calculation method of Karpinski and Steinman (39). Responses (< 350 ms) and non-responses (more than 1,500 ms) were eliminated, and incorrect responses were replaced with the block mean plus an error penalty of 400 ms. For each individual, the average response time for the compatibility task (disgust phrase–bad words) was subtracted from the average response time for the incompatibility task (disgust phrase–good words), and the difference was divided by the standard deviation of all correct RTs for both the compatible and incompatible tasks. This quotient is referred to as the *D*-score. Positive *D*-scores indicated that participants implicitly considered the disgusting phrase negative, while negative *D*-scores indicated that participants implicitly considered the disgusting phrase positive. Higher *D*-scores refer to more negative attitudes toward disgusting phrases.

All data analyses were performed using SPSS 22.0 software. *D*-scores, the average response times (RTs), and accuracy were analyzed using a 3 (cycle phase: menses vs. follicular vs. luteal) × 2 (disgust type: pathogen disgust vs. moral disgust) × 2 (task type: compatible vs. incompatible) mixed analysis of variance (ANOVA), respectively. When the assumption of sphericity did not hold, the degrees of freedom of the *F* value ratio was corrected using Greenhouse–Geisser. Bonferroni corrections were performed for *post-hoc* testing of significant main effects. Simple effect analyses were performed to test for significant interactions. Descriptive statistics are expressed as the mean ± standard deviation (*M* ± SD). The significance level of all statistical analyses was set to 0.05, and the effect size of all variance analysis was reported by partial eta-squared (η*p*^2^), with 0.01, 0.06, and 0.14 representing a small, medium, and large effect size (42), respectively. For the explicit intensity assessment, in the analysis of response duration, trials with RTs >4,000 ms and < 500 ms were excluded. A similar ANOVA was performed on the disgust intensity scores and response times.

#### 2.1.5. Results

##### 2.1.5.1. SC-IAT

**Figure 2:**
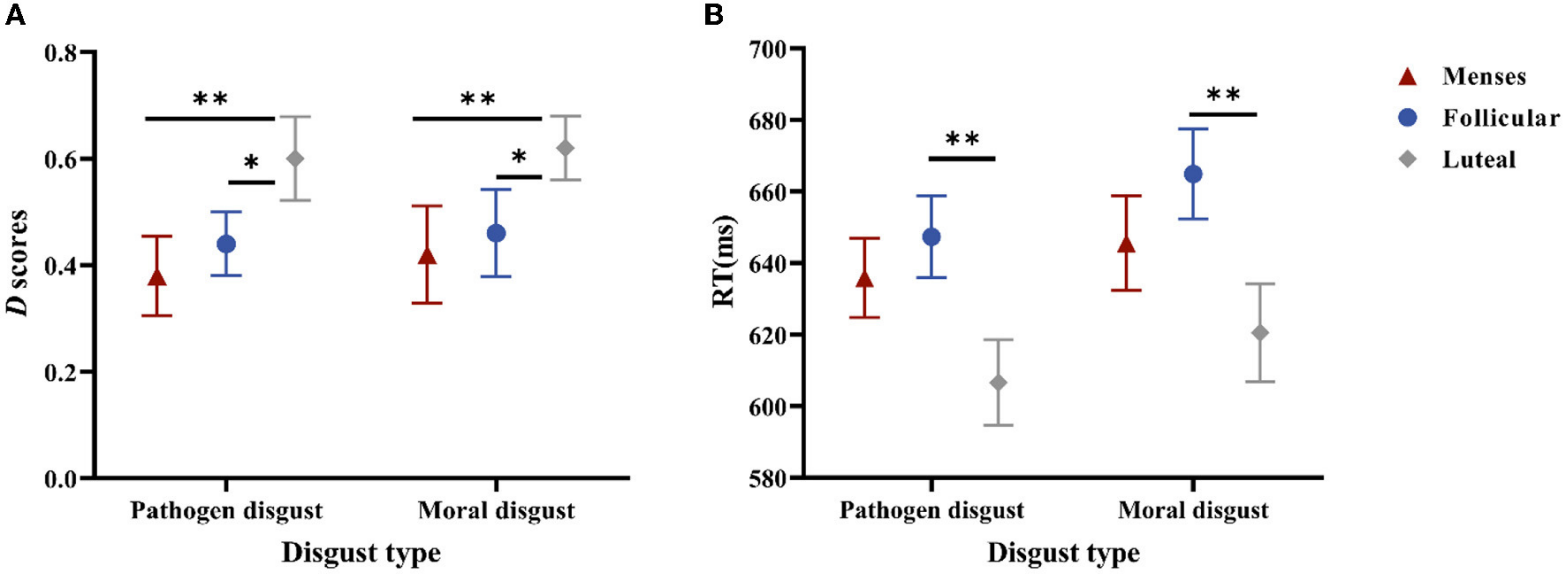
Performances in the SC-IAT. **(A)** is the *D* scores for women as a function of disgust type ^*^ cycle phase. **(B)** is the mean RTs of the incongruent and congruent responses for women as a function of disgust type ^*^ cycle phase. Error bars represent the standard error of the mean. ^**^p < 0.01; ^*^p < 0.05.

Regarding *D*-scores, a significant main effect of the cycle phase was confirmed, *F*_(2, 85)_ = 4.332, *p* = 0.016, and η^2^ = 0.093. The *post-hoc* paired *t*-tests indicated that the *D*-scores in the luteal phase (*M* = 0.61, *SE* = 0.05) were higher than those in the menses phase (*M* = 0.40, *SE* = 0.05, and *p* < 0.006) or the follicular phase (*M* = 0.45, *SE* = 0.05, and *p* < 0.038) but did not differ between the menses phase and follicular phase (*p* = 0.475). In addition, the main effect of disgust type and the interaction effect of cycle phase × disgust type were not significant (*p* = 0.631 and *p* = 0.995, respectively) (see Figure 2A).

Regarding RTs, the main effect of the cycle phase was significant, *F*_(2, 85)_ = 4.023, *p* = 0.021, and η^2^ = 0.086; RTs in the luteal phase (*M* = 613.51, *SE* = 11.60) were shorter than those in the follicular phase (*M* = 656.12, *SE* = 9.73, and *p* = 0.006), but RTs did not differ between the menses phase (*M* = 640.74, *SE* = 10.93) and follicular phase (*p* = 0.32) or between the menses phase and luteal phase (*p* = 0.077). The main effect of disgust type was significant, *F*_(1, 85)_ = 3.987, *p* = 0.049, and η^2^ = 0.045, with pathogen disgust RTs (*M* = 629.89, *SE* = 6.63) being shorter than moral disgust RTs (*M* = 643.69, *SE* = 7.60). The main effect of task type was also significant, *F*_(1, 85)_ = 194.37, *p* < 0.001, and η^2^ = 0.696, with the compatible task RTs (*M* = 602.81, *SE* = 6.45) being shorter than those of the incompatible task (*M* = 670.78, *SE* = 6.94). However, no significant interaction effects were observed (*p*s > 0.1) (see Figure 2B).

Regarding accuracy, the main effect of disgust type was significant, *F*_(1, 85)_ = 5.211, *p* = 0.025, and η^2^ = 0.058, with the accuracy of the pathogen disgust being higher (*M* = 96.69, *SE* = 0.37) compared to that of the moral disgust (*M* = 95.39, *SE* = 0.55). The main effect of the task type was significant, *F*_(1, 85)_ = 50.301, *p* < 0.001, and η^2^ = 0.372, with the accuracy of the compatible task being higher (*M* = 97.77, *SE* = 0.28) compared to that of the incompatible task (*M* = 94.31, *SE* = 0.56). In addition, the main effect of the cycle phase was not significant, and there were no significant interaction effects (*p*s > 0.1).

##### 2.1.5.2. Explicit measures

For the disgust intensity score, the main effect of disgust type was significant, *F*_(2, 84)_ = 1065.5, *p* < 0.001, and η^2^ = 0.958, and *post-hoc* paired *t*-tests revealed that the intensity score for the pathogen disgust phrase (*M* = 7.04, *SE* = 0.11) was higher than those for the moral disgust (*M* = 6.36, *SE* = 0.14, *p* < 0.001) and neutral (*M* = 1.52, *SE* = 0.05, and *p* < 0.001) phrases; the intensity score for the moral disgust phrases was higher than that for the neutral phrases (*p* < 0.001). However, the main effect of the cycle phase and the interaction effect of cycle phase × disgust type were not significant (*p* = 0.385; *p* = 0.081).

Further analysis suggests that stimulus intensity affects the perception of disgust intensity during the cycle phase and that high stimulus intensity masks fluctuations in disgust perception during the cycle phase (more details are provided in Supplementary material). Therefore, we selected only the low-intensity group for further analysis. In the low-intensity stimuli, 3 (cycle phase: menses vs. follicular vs. luteal) × 3 (disgust type: pathogen disgust vs. moral disgust vs. neutral) mixed ANOVA was used to determine the effect of cycle phase and disgust type on disgust intensity score.

**Figure 3:**
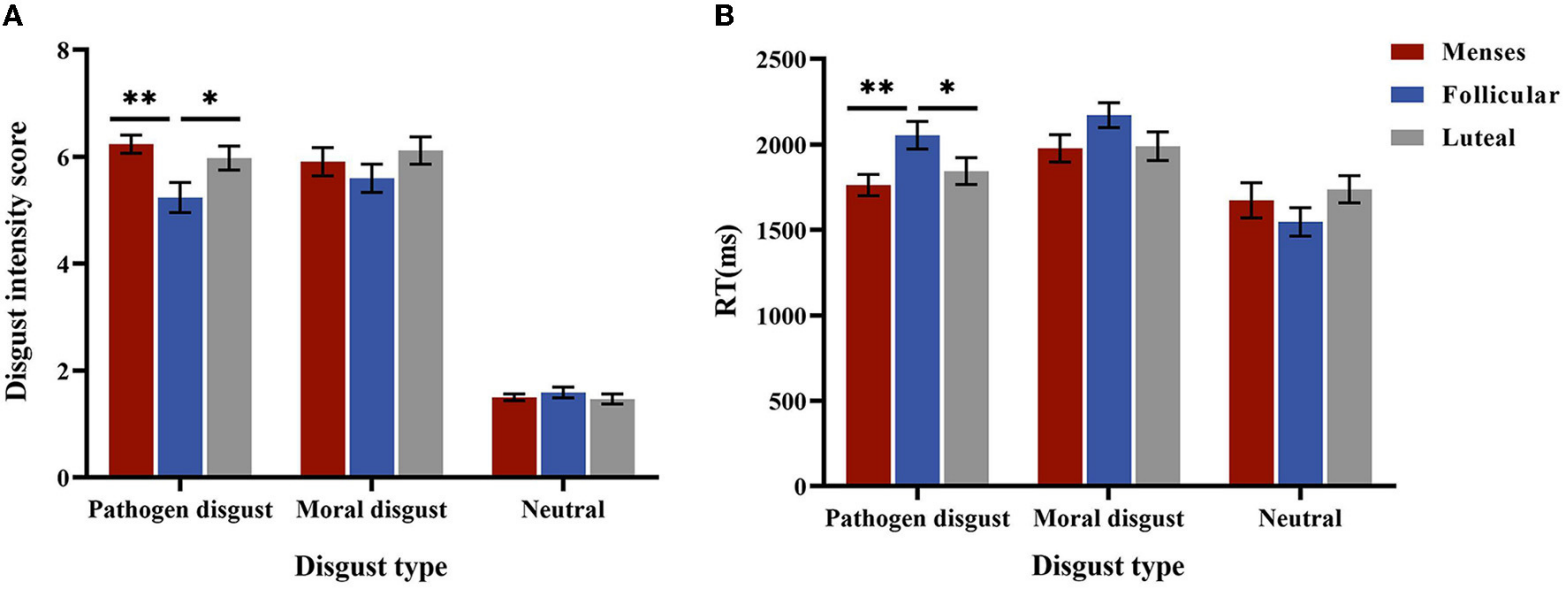
Performances in the explicit intensity score. **(A)** is the disgust intensity score for women as a function of disgust type ^*^ cycle phase in low-intensity stimulus. **(B)** is the mean RTs for women as a function of disgust type ^*^ cycle phase. Error bars represent the standard error of the mean. ^**^p < 0.01; ^*^p < 0.05.

The interaction effect of cycle phase × disgust type was significant, *F*_(4, 170)_ = 2.939, *p* = 0.029, and η^2^ = 0.061. For the pathogen disgust phrase, the intensity scores in the menses (*M* = 6.24, *SE* = 0.23) and luteal (*M* = 5.98, *SE* = 0.23) phases were higher than that in the follicular phase (*M* = 5.60, *SE* = 0.26, *p*= 0.003*; p*= 0.025). However, the moral disgust and neutral phrases did not differ in intensity scores (*ps* > 0.1). The main effect of disgust type was significant, *F*_(2, 84)_ = 431.15, *p* < 0.001, and η^2^ = 0.913, with the intensity score of the pathogen disgust phrases (*M* = 5.82, *SE* = 0.13) and moral disgust phrases (*M* = 5.97, *SE* = 0.15) being higher than that of the neutral phrases (*M* = 1.58, *SE* = 0.06, *p*s < 0.001). The main effect of the cycle phase was not significant (*p* = 0.096) (see Figure 3A).

For RTs, the interaction between the cycle phase and disgust type was significant, *F*_(4, 170)_ = 4.758, *p* = 0.001, and η^2^ = 0.101. A simple effect analysis showed that RTs for the pathogen disgust phrases were longer in the follicular phase (*M* = 2054.51, *SE* = 75.21) than in the menses (*M* = 1762.90, *SE* = 75.21, *p* = 0.007) and luteal (*M* = 1843.81, *SE* = 73.95, *p* = 0.049) phases but did not differ among the cycle phases for moral disgust and neutral phrases (*p* > 0.1). The main effect of disgust type was significant, *F*_(2, 84)_ = 52.581, *p* < 0.001, and η^2^ = 0.556, with RTs for the neutral phrases (*M* = 1652.53, *SE* = 51.61) being shorter than those for the pathogen disgust (*M* = 1887.07, *SE* = 43.18) and moral disgust (*M* = 2046.91, *SE* = 45.52, *p*s < 0.001) phrases. Moreover, the pathogen disgust phrase was associated with shorter RTs than the moral disgust phrase (*p* < 0.001). The main effect of the menstrual cycle was not significant (*p* = 0.504) (see Figure 3B). Because the two-way interaction between stimulus intensity and cycle phase or between disgust type and cycle phase was not significant (*ps* > 0.05), we did not perform further analyses.

### 2.2. Experiment 2

#### 2.2.1. Participants

A total of 25 healthy female undergraduates completed three sessions of the rs-fMRI. The subjects were selected based on the same criteria used in Experiment 1. The menstrual group was scanned during cycle days 2–5, the follicular group was scanned during cycle days 10–13, and the luteal group was scanned 3–7 days before the next menstruation. To control for a possible session effect, the testing order was randomized across subjects such that all three cycle phases were equally distributed across the three time points. Seven participants were scanned but later excluded due to menstrual irregularities. After data pre-processing, an additional three subjects were excluded because of excessive head motion (>4 mm or 4°). A total of 15 subjects, aged 18–21 with a mean age of 19.56 ± 0.98 years, were finally included in the analysis. The study was conducted in accordance with the Declaration of Helsinki and reviewed by the local ethics committee of Sichuan Normal University.

#### 2.2.2. Procedures

Testing sessions were conducted in the afternoon between 2:00 p.m. and 6:30 p.m. One day before each visit, the participants were instructed to abstain from alcohol and caffeine consumption within 12 h. Brushing teeth and smoking within 3 h of the beginning of the experiment were also prohibited. Furthermore, they were requested not to eat a meal or consume dairy products within 1 h of the experiment. Upon arrival, participants provided no < 1 ml of saliva for the hormone assay. Saliva samples were stored in polypropylene centrifuge tubes and immediately frozen in a refrigerator at −20°C. Next, participants completed several self-report questionnaires: the Beck Depression Inventory (BDI), the State Anxiety Inventory (S-AI), and the Eysenck Personality Questionnaire-Revised Short Scale for Chinese (EPQ-RSC) (for details of questionnaire materials, see Supplementary material). The scale scores of all participants in the data analysis phase were within three standard deviations to avoid confounding effects. Finally, all participants completed an 8-min resting-state scan.

#### 2.2.3. Magnetic resonance imaging data acquisition

A Siemens 3.0T Trio scanner (Siemens, Erlangen, Germany) was used for the MRI data acquisition. High-resolution T1 structural images were acquired using a magnetization-prepared rapid gradient echo (MPRAGE) sequence with the following parameters: repetition time (TR) = 2,530 ms; echo time (TE) = 2.98 ms; flip angle = 7°; axial slices = 192 layers; thickness = 1.0 mm; field of view (FOV) = 256 × 256 mm^2^; resolution matrix = 64 × 64; and voxel size = 0.5 × 0.5 × 1 mm3. Functional images were obtained using a planar echo-imaging pulse sequence (echo-planar imaging) (EPI) with the following parameters: TR, 2,000 ms; TE, 30 ms; flip angle, 90°; axial slices, 62 layers; slice thickness, 2 mm; FOV, 224 mm × 224 mm; resolution matrix = 64 × 64; and voxel size = 2 × 2 × 2 mm^3^.

#### 2.2.4. Data analysis

##### 2.2.4.1. Hormonal analyses of the saliva samples

All samples were collected and sent to Shenzhen Boaokang Biotechnology Co., Ltd., for analysis. On the day of the hormone assay, the samples were thawed and centrifuged at 10,000 × g for 10 min at 4°C, and the supernatants were collected for the assay. A competitive enzyme-linked immunosorbent assay (c-ELISA) was used to detect estradiol and progesterone in the saliva samples using estradiol (NO.DESLV4188) and progesterone (NO.DESLV5911) kits provided by Demeditec, Germany. The detection range of the estradiol kit is 1–100 pg/ml and sensitivity is 0.6 pg/ml; the detection range of the progesterone kit is 10–5,000 pg/ml and sensitivity is 5 pg/ml. One-way ANOVA was used to calculate the estradiol and progesterone levels in different cycle phases (menses, follicular, and luteal).

##### 2.2.4.2. fMRI data analysis

Imaging data analysis was performed using the Conn toolbox (http://www.nitrc.org/projects/conn) version 20b and SPM12 (http://www.fil.ion.ucl.ac.uk/spm) running on MATLAB R2018b (MathWorks, Natick, MA, USA). Pre-processing of rs-fMRI images included slice-time correction, realignment and unwarping, segmentation of gray matter, white matter, and CSF, normalization to the Montreal Neurological Institute template, and spatial smoothing based on a Gaussian kernel set at 8-mm full width at half maximum. The artifact detect based scrubbing method, implemented in Conn, was further used to detect outlying volumes with high motion (using a 2-mm subject motion threshold and a global signal threshold set at *Z* = 9). Nuisance variable regression was then performed, and the first five principal components from the segmented white matter and CSF were regressed out of the signal. The six motion realignment parameters and their first-order derivatives, quadratic effects, and outlier volumes detected during the scrubbing procedure were similarly regressed out of the signal. Additional steps included linear detrending and bandpass filtering the residual signals at 0.008–0.09 Hz.

The regions of interest (ROIs) were selected based on a large body of literature describing them as core nodes of the corresponding networks such as the bilateral insula and anterior cingulate cortex for the SNs (33, 43). We examined the bilateral amygdala, insula, and anterior cingulate and paracingulate gyri (ACG) RSFC with AAL atlas-defined ROIs through the seed-based functional connectivity analysis using the CONN toolbox (44) (see an example of ROI in Supplementary material). The group-level amygdala, insula, and ACG RSFC were determined using one-sample *t*-tests. Differences across cycles were examined using paired *t*-tests. To balance the risk of type I and II errors, voxels were considered significant if they survived a cluster-level false discovery rate (FDR) correction of *p* < 0.05 after thresholding at an uncorrected voxel level of *p* < 0.001.

#### 2.2.5. Results

##### 2.2.5.1. Hormone levels

For estradiol, one-way ANOVA revealed a significant main effect of the cycle phase, *F*_(2, 34)_ = 3.845, *p* = 0.031, and η^2^ = 0.184. Follow-up analyses of the cycle phase indicated that the estradiol level was significantly lower during menses (mean = 4.87 pg/mL, SE = 0.39) compared with the follicular (mean = 6.75 pg/mL, SE = 0.86; *p* < 0.001) and the luteal (mean = 6.33 pg/mL, SE = 0.52; *p* < 0.001) phases but did not differ between the follicular and luteal phases (*p* > 0.05).

For progesterone, one-way ANOVA revealed a significant main effect of cycle phase *F*_(2, 34)_ = 11.71, *p* = 0.003, and η^2^ = 0.408. Follow-up analyses of the cycle phase indicated that progesterone levels were significantly higher during the luteal phase (mean = 103.99 pg/mL, SE = 23.58) compared with the menses (mean = 22.41 pg/mL, SE = 3.57; *p* < 0.001) and the follicular (mean = 20.25 pg/mL, SE =2.29; *p* < 0.001) phases but did not differ between the menses and follicular phase (*p* > 0.05).

##### 2.2.5.2. Amygdala and salience network connectivity

**Figure 4:**
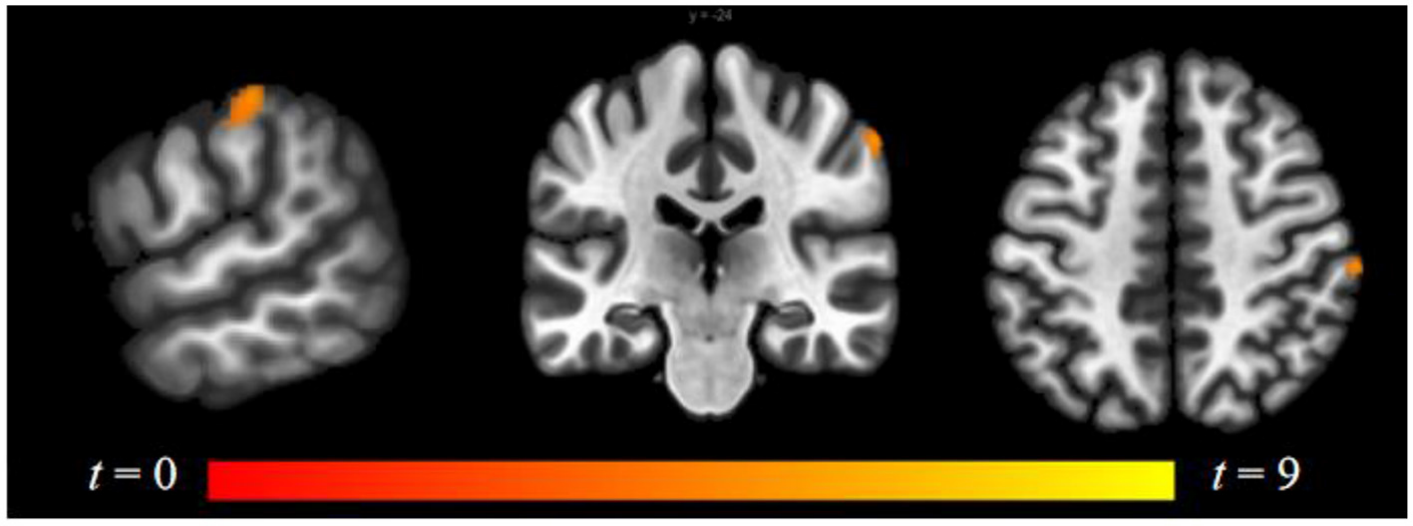
Within-group differences between menses phase and follicular phase. The hot color scale indicates higher left amygdala–right supramarginal gyrus resting-state functional connectivity (RSFC) in the menses phase than follicular phase. Coordinates are given in MNI space and statistics in *t*-values.

Compared to follicular women, menses women showed increased connectivity between the amygdala and supramarginal gyrus (see Figure 4 and Table 2). Follicular women did not show any regions of increased connectivity relative to menses women in the amygdala, insula, or ACG.

**Table 2:** Differences in amygdala, ACG, and insula resting-state functional connectivity for naturally cycling women.

		**x**	**Y**	**z**	***t*-Score**	**Cluster size**
Menses phase > follicular phase						
Left amygdala						
Right supramarginal gyrus		+62	−24	+48	5.04	43
Luteal phase > follicular phase						
Left insula						
Precuneous		−18	−54	+12	5.44	46
Right lingual gyrus		+8	−58	+4	6.46	77
Left lingual gyrus		−12	−54	−8	6.31	38
Right insula						
Precuneous		+12	−58	+68	7.86	21
Left lateral occipital cortex		−30	−78	+32	6.22	45
ACG						
Left lingual gyrus		−12	−52	−6	7.38	36
Follicular phase > luteal phase						
Right insula						
Right frontal pole		+16	+52	+32	6.09	54
Left middle temporal gyrus		−52	−10	−24	6.52	27
Right middle temporal gyrus		+52	−2	−28	5.94	33

All clusters are significant at p < 0.001. False discovery rate (FDR) corrected for multiple comparisons. ACG, anterior cingulate and paracingulate gyri.

**Figure 5:**
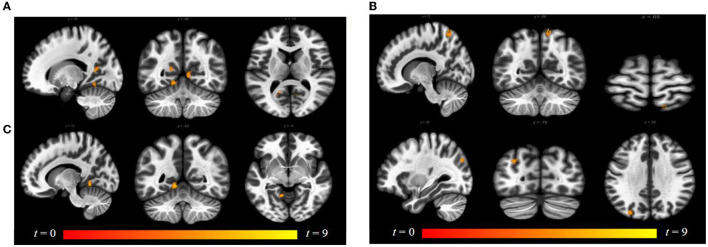
Within-group differences between luteal phase and follicular phase. The hot color scale indicates **(A)** higher left insula–precuneus, right and left lingual gyrus resting-state functional connectivity (RSFC) in the luteal than follicular phase, as well as increased **(B)** right insula–precuneus, lateral occipital cortex, and **(C)** ACG–left lingual gyrus. Coordinates are given in MNI space and statistics in *t*-values. ACG, anterior cingulate and paracingulate gyri.

**Figure 6:**
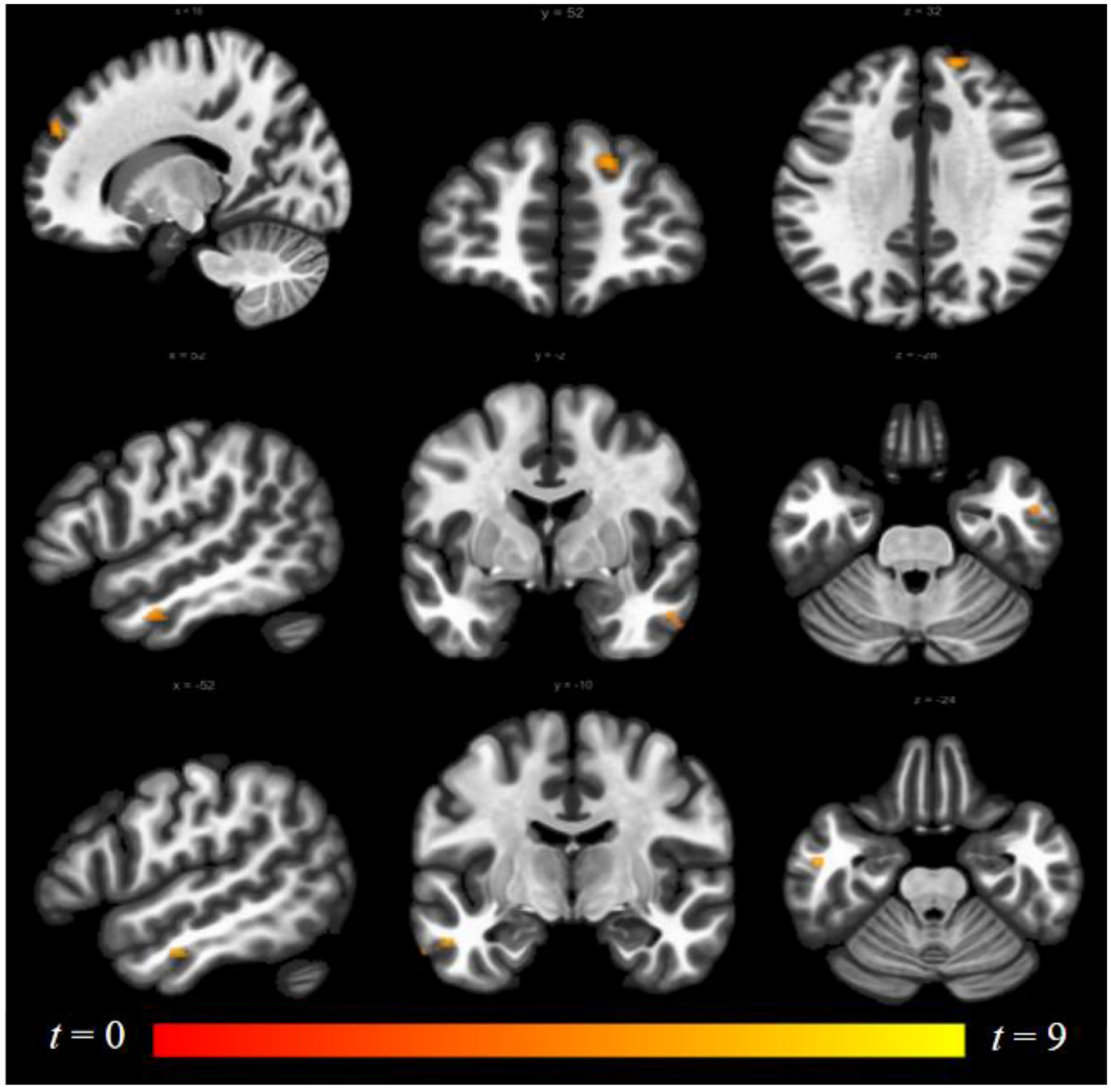
Within-group differences between follicular phase and luteal phase. The hot color scale indicates higher right insula–right frontal pole, left and right middle temporal gyrus resting-state functional connectivity (RSFC) in the follicular than luteal phase. Coordinates are given in MNI space and statistics in *t*-values.

Luteal women showed increased connectivity between the insula and precuneus, lingual gyrus, and lateral occipital cortex and increased connectivity between the ACG and lingual gyrus compared to follicular women (see Figure 5 and Table 2). Follicular women also showed increased connectivity between the insula and frontal pole and the middle temporal gyrus compared to luteal women (see Figure 6 and Table 2).

## 3. Discussion

This study was the first to explore how ovarian hormones affect pathogen and moral disgust processing across the menstrual cycle using both implicit and explicit tasks. Furthermore, it investigated the effects of the menstrual cycle on the amygdala and salience network resting-state functional connectivity. The behavioral results showed that women during the luteal phase had higher *D*-scores and shorter RTs to disgust stimuli (for both the pathogen and moral disgust stimuli) compared to the menses and follicular phases, suggesting that women in the luteal phase had more negative implicit attitudes toward the disgust stimuli. In contrast, women during the follicular phase had fewer feelings of disgust and longer RTs to pathogen stimuli compared to the menses and luteal phases, but this effect was moderated by the intensity of the stimuli. The rs-fMRI study showed that women during the luteal phase had higher functional connectivity in the insula and ACG than those in the follicular phase. Compared to the menstrual phase, women had lower functional connectivity in the amygdala during the follicular phase. Altogether, the more negative attitude to disgust stimuli and the enhanced functional connectivity of the salience network during the luteal phase may be associated with high progesterone levels, whereas lower disgust feelings and reduced functional connectivity of the amygdala during the follicular phase may be associated with high estradiol levels.

First, in line with our hypotheses, women in the luteal phase had more negative implicit attitudes and higher sensitivity toward disgust stimuli for both the pathogen and moral disgust stimuli. This is consistent with the findings of Miłkowska et al. (21) and Zelazniewicz et al. (22) and supports the CPH. Progesterone in the luteal phase increases feelings of disgust and activates the behavioral immune system to reduce the risk of infection (20–22). The result of explicit measures of the disgust intensity task revealed that only menstrual cycle effects in the low-intensity group may be due to the high intensity of the disgust stimuli causing a ceiling effect on the subjects' ratings, thus masking the effect of the menstrual cycle (45, 46). However, most researchers have focused on the difference in feelings of disgust between the luteal phase with high progesterone levels and the other two phases, while few have directly compared the menses and follicular phases.

In this study, we found that the intensity of pathogen disgust was significantly higher in the menses phase than in the follicular phase in the low-intensity group. The reason for this result may be the suppressive effect of high estradiol in the follicular phase on negative emotions. The results of Gasbarri et al. (26) showed higher error rates in the processing of disgust expressions in women during the follicular phase compared to the menses phase. Kamboj (27) further demonstrated that the decrease in disgust expression recognition during the follicular phase may be related to estradiol levels. In addition, women reported higher subjective wellbeing during the follicular phase and more negative emotions during menses (47). These findings also support a decrease in the intensity of feelings of disgust during the follicular phase, and the intrinsic physiological mechanism may be the suppressive effect of estradiol.

For moral stimuli, after excluding the effect of intensity, there was still no detectable effect on the menstrual cycle, which is also in line with previous findings using self-reports (21, 46). Notably, Molho et al. (48) thought that moral disgust meant not only avoiding social norm violations but also referring to less costly indirect aggression toward and punishment of others (e.g., gossip), which may influence individuals' explicit ratings.

Second, we found higher functional connectivity in the salience network during the luteal phase (31, 32), as reflected in the increased insula and ACG RSFC in the luteal phase compared to those in the follicular phase. The salience network plays an important role in detecting salient stimuli and integrating the sensory, emotional, and cognitive processes (32, 43), with higher progesterone levels during the luteal phase and results in greater sensitivity to threatening stimuli. Alterations in the connectivity in SNs have been reported more frequently in affective disorders. For example, increased connectivity between salience nodes in post-traumatic stress disorder (PTSD) is believed to represent hypervigilance and increased sensitivity to threats (49). Andreano et al. (31) proposed a luteal window of the vulnerability model in which a surge in progesterone levels during the luteal phase and enhanced functional brain connectivity are associated with increased stress responses and better memory for unpleasant, exciting events, thereby leading to increased negative emotions and susceptibility to emotional disturbances.

However, although ample evidence supports increasing amygdala activity in women with the premenstrual dysphoric disorder (PMDD) during the luteal phase in a meta-analysis (50), the current investigation shows no increase in amygdala activity during the luteal phase in naturally cycling women. Gingnell et al. (51) noted that luteal phase-related increases in amygdala reactivity were found only in individuals with high anxiety traits. In addition, Petersen et al. (52) found no differences in amygdala activation between healthy controls and PMDD groups across menstrual phases. Amygdala connectivity reductions during luteal vs. follicular stages were observed in healthy controls and women with PMDD, and they appear to reflect normal cyclicity in brain connectivity (53). However, we found an attenuation of amygdala activity in the presence of higher levels of estradiol (i.e., menses vs. follicular differences). Goldstein et al. (35) provided the first evidence that estradiol may attenuate female arousal through cortical–subcortical control in the hypothalamic–pituitary–adrenal axis (HPA) circuit. Subsequently, Albert et al. (54) found that women with high estradiol levels showed significantly less deactivation in limbic regions and had less subjective distress during psychosocial stress compared to women with low estradiol levels; in other words, high estradiol levels attenuated brain activation changes and negative mood responses to psychosocial stress.

This study has several limitations. First, we selected only female undergraduates and a relatively small sample size, which may limit the generalizability of our results. Another important limitation is that hormone assay data were not collected to confirm the menstrual cycle phase in the behavioral task. Although only women who were able to report the start date of their last three menstrual cycles were enrolled in the present study, the use of hormone assay data would allow for greater confidence in the estimation of the menstrual cycle phase. In addition, recent studies have found that olfactory or gustatory stimuli are more direct and effective in inducing disgust in subjects than phrases or pictures (55–57). Future studies should consider other age groups, such as adolescents (who have the most dramatic hormonal fluctuations) and menopausal women and further combine task-fMRI to explore the effects of ovarian hormones on disgust emotion in different sensory channels.

## 4. Conclusion

This study is the first of its kind to investigate how ovarian hormones affect pathogen and moral disgust processing across the menstrual cycle using both implicit and explicit tasks. The more sensitive and negative attitude toward disgust stimuli shown during the luteal phase may be associated with high progesterone levels, whereas the inhibition of disgust stimuli shown during the follicular phase may be associated with high estradiol levels. Estradiol and progesterone may have opposite effects on disgust processing, with estradiol decreases disgust while progesterone enhances disgust. This might help young women fall in love easily while pregnant women avoid toxic materials.

In addition, disgust is a core survival emotion that makes us expel something toxic to us, and it is one of the most prototypical emotions (58). Like what we proposed the “Three Primary Color Model of Basic Emotions” (3, 38), we hypothesized that disgust might be one of the three most primary emotions (joy, disgust, and fear) like the three primary colors. However, it is the least studied emotion; however, it has recently been found to be the major cause of most affective disorders. The better understanding of the relationship between ovarian hormones and disgust processing provided by this study's findings may provide a new perspective on how to relieve affective and premenstrual dysphoric disorders.

## Data availability statement

The raw data supporting the conclusions of this article will be made available by the authors, without undue reservation.

## Ethics statement

The studies involving human participants were reviewed and approved by Committee of Sichuan Normal University. The patients/participants provided their written informed consent to participate in this study.

## Author contributions

XZ, FW, and SG contributed to the conception and design of the study. ML and XZ organized the database and performed the statistical analysis. ML wrote the first draft of the manuscript. ZH, YL, and BZ wrote sections of the manuscript. All authors contributed to the manuscript revision, and read and approved the submitted version.
