# Supplementary material for: Opposite effects of estradiol and progesterone on woman's disgust processing
Source: Front Psychiatry. 2023 Apr 5;14:1161488. doi: 10.3389/fpsyt.2023.1161488 (PMC10115175; doi:10.3389/fpsyt.2023.1161488)
Supplement: Supplementary file 1 [file Data_Sheet_1.docx]

Supplementary Material

Opposite effects of estradiol and progesterone on woman’s disgust processing

**Mei Liu^1#^, Xia Zhang^1#^, Zhengming He^1#^, Yuan Liang^1^, Bihong Zou^1^, Xianjun Ma^3^*, Simeng Gu^1,2^*, Fushun Wang^1^***

* **Correspondence** and requests for materials should be addressed to: Xianjun Ma maxianjun@126.com; Fushun Wang (13814541138@163.com) or Simeng Gu ([gsm_2007@126.com](mailto:gsm_2007@126.com)).

# Supplementary Data

**Evaluation of emotion-inducing materials**

In the present study, we chose to use text-based phrase stimuli to induce disgust for three reasons: first, picture and video disgust stimuli usually easily induce high-intensity pathogen disgust feelings, leading to the ceiling effects; Besides that, picture and video stimuli are difficult to properly present moral disgust, because many stimuli that can induce moral disgust are behavioral or some quality feature. In contrast to pictures and videos, phrase stimuli can present both different types of disgust stimuli well and induce different intensities of disgust by adding verbs (e.g., see feces, touch feces). Text-based emotional phrase stimulus to reliably induce emotional experiences has been well demonstrated in previous studies(1-3) .

The disgust and neutral phrases were carefully selected in two separate pilot studies. First, using the self-assessment manikin (SAM) method, 30 participants were asked to assess the degree of moral violation (weak to very strong), arousal (very calm to very exciting), valence (very unpleasant to very pleasant), intensities (weak to very strong), and familiarity (very unfamiliar to very familiar) of 220 Chinese phrases on a 9-point Likert-type scale. They were then placed into different emotional categories: happy, fearful, sad, disgusted, angry, and neutral. Thirty pathogen disgust phrases (e.g., touch feces), 30 moral disgust phrases (e.g., drug abuse), and 30 neutral phrases (e.g., phone calls) were selected according to the methods of Moll et al. (2) and Scott et al. (4) :

(1) to be considered a pathogen disgust phrase, more than 80% of the participants had to have placed them into the “disgust” category, and disgust phrases were only included if they received the degree of moral violation lower than 3, a valence rating lower than 4 and the familiarity rating higher than 5;

(2) to be considered a moral disgust phrase, more than 80% of the participants had to have placed them into the “disgust” category, and moral disgust phrases were only included if they received the degree of moral violation rating higher than 4, a valence rating lower than 4 and the familiarity rating higher than 5.

(3) to be considered a neutral phrase, more than 80% of the participants had to have placed them into the neutral category, and neutral phrases were only included if they received the degree of moral violation lower than 3, a valence rating lower than 5 and the familiarity rating higher than 5.

**Table S1** Descriptive statistics (M±SD) of various stimuli attributes

|  | Moral violation intensity | | Disgust intensity | Valence | Arousal | Familiarity |
| --- | --- | --- | --- | --- | --- | --- |
| Pathogen disgust | | 1.97 ± 0.67 | 5.83±1.00 | 2.80±0.57 | 6.32±0.63 | 7.27±0.30 |
| Moral disgust | | 4.88±1.20 | 5.84±0.75 | 3.23±0.59 | 5.97±0.59 | 7.76±0.19 |
| Neutral control | | 1.05±0.14 | 1.06±0.11 | 5.16±0.48 | 4.59±1.00 | 8.20±0.17 |

**Questionnaires**

The Beck Depression Inventory (BDI): The Chinese version of the Baker Depression Inventory is a 21-item questionnaire designed to assess the degree of depressive symptoms regarding the previous week (5). Each item is rated on a Likert four-point Likert-type scale ranging from "none" (0 points) to "extremely severe" (3 points), with higher scores indicating more severe depressive symptoms (5).

State Anxiety Inventory (S-AI): Revised Chinese version of the State-Trait Anxiety Inventory-Form Y (STAI-Y) was used to measure anxiety using a 4-point Likert-type scale for each item ranging from "nothing at all" (1 point) to "very obvious" (4 points). It is structured by the factors "state anxiety present" and "state anxiety absent”. The higher the total score, the higher the individual's state anxiety level (6).

Eysenck Personality Questionnaire Chinese Version (EPQ-RSC): A study indicated that the influence of the menstrual cycle on the emotional processing of healthy women of childbearing age was regulated by the level of neuroticism. The Scale Neuroticism has satisfactory reliability, used to measure the neuroticism level, with higher scores indicating more unstable emotions such as anxiety, tension, and irritability (7).

# Supplementary Figures

**Concerning the stimuli’s intensity effects**

3 (cycle phase: menses vs. follicular vs. luteal) × 2 (disgust type: pathogen disgust vs. moral disgust) × 3(stimulus intensity: low vs. medium vs. high), A mixed ANOVA was used to determine the effect of cycle phase, disgust type and stimulus intensity on disgust intensity score. The interaction effect of cycle phase × stimulus intensity was significant, *F*(4,170) = 2.973, *p* = 0.021, *η*^2^ = 0.065. In the low-intensity of stimulus, the disgust intensity score in the menstrual phase (*M* = 6.07, *SE* = 0.22) and luteal phase (*M* = 6.05, *SE* = 0.22) was higher than follicular phase (*M* = 5.42, *SE* = 0.22, *p* < 0.05). But in the medium and high level, there were no significant effects among cycle phase (*p* > 0.1). The interaction effect of stimulus intensity × disgust type was significant, *F*(2,170) = 73.401, *p* < 0.001, η2 = 0.463. the disgust intensity score of pathogen disgust on low intensity (M = 5.82, SE = 0.13) was not different from moral disgust (M = 5.87, SE = 0.15, *p* = 0.649), but the intensity score of pathogen disgust was higher than moral disgust on medium and high intensity (*p*s < 0.001). the main effect of disgust type was significant, *F*(1,85) = 45.349, *p* < 0.001, *η*^2^ = 0.348, with intensity score of pathogen disgust(*M* = 7.04, *SE* = 0.11) higher than moral disgust(*M* = 6.36, *SE* = 0.14). The main effect of stimulus intensity was significant, *F*(2,170) = 428.015, *p* < 0.001, *η*^2^ = 0.834, but the main effect of the cycle phase was not significant (*p* = 0.283).

**Figure S1** Performances as a function of cycle phase * intensity of stimulus in the disgust intensity score. In low intensity of stimulus, the disgust intensity score in the mense phase and luteal phase was higher than follicular phase. (*p*s < 0.05)


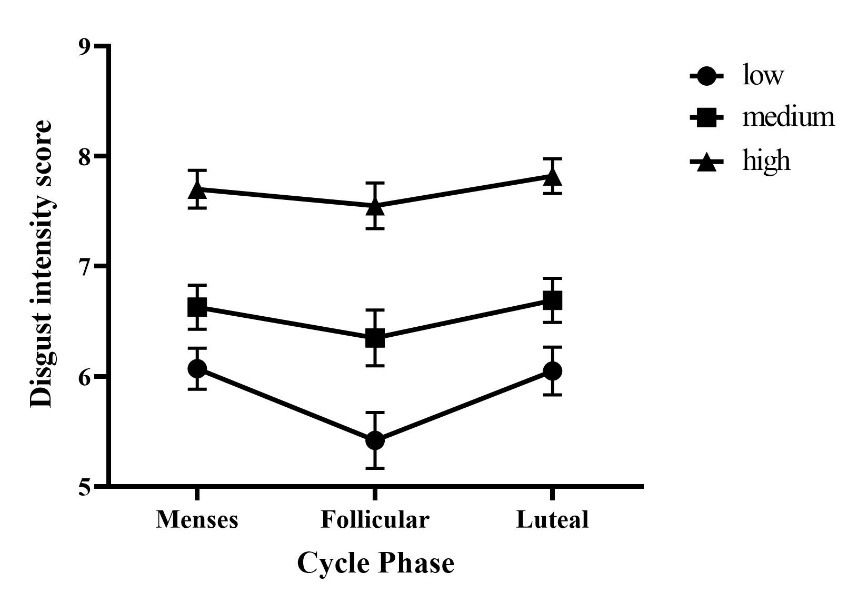


**Selecting the regions of interest (ROIs)**

The ROIs were selected based on a large body of literature describing them as core nodes of the corresponding networks such as bilateral AI and ACC for the SN (8-10).

We examined the bilateral amygdala, insula, and anterior cingulate and paracingulate gyri (ACG) RSFC with AAL atlas-defined regions of interest (ROIs) (8, 11) through seed-based functional connectivity analysis using the CONN toolbox (12). And we facilitate BrainNet Viewer (http://www.nitrc.org/projects/bnv/) to create an example of ROIs as supplementary materials.


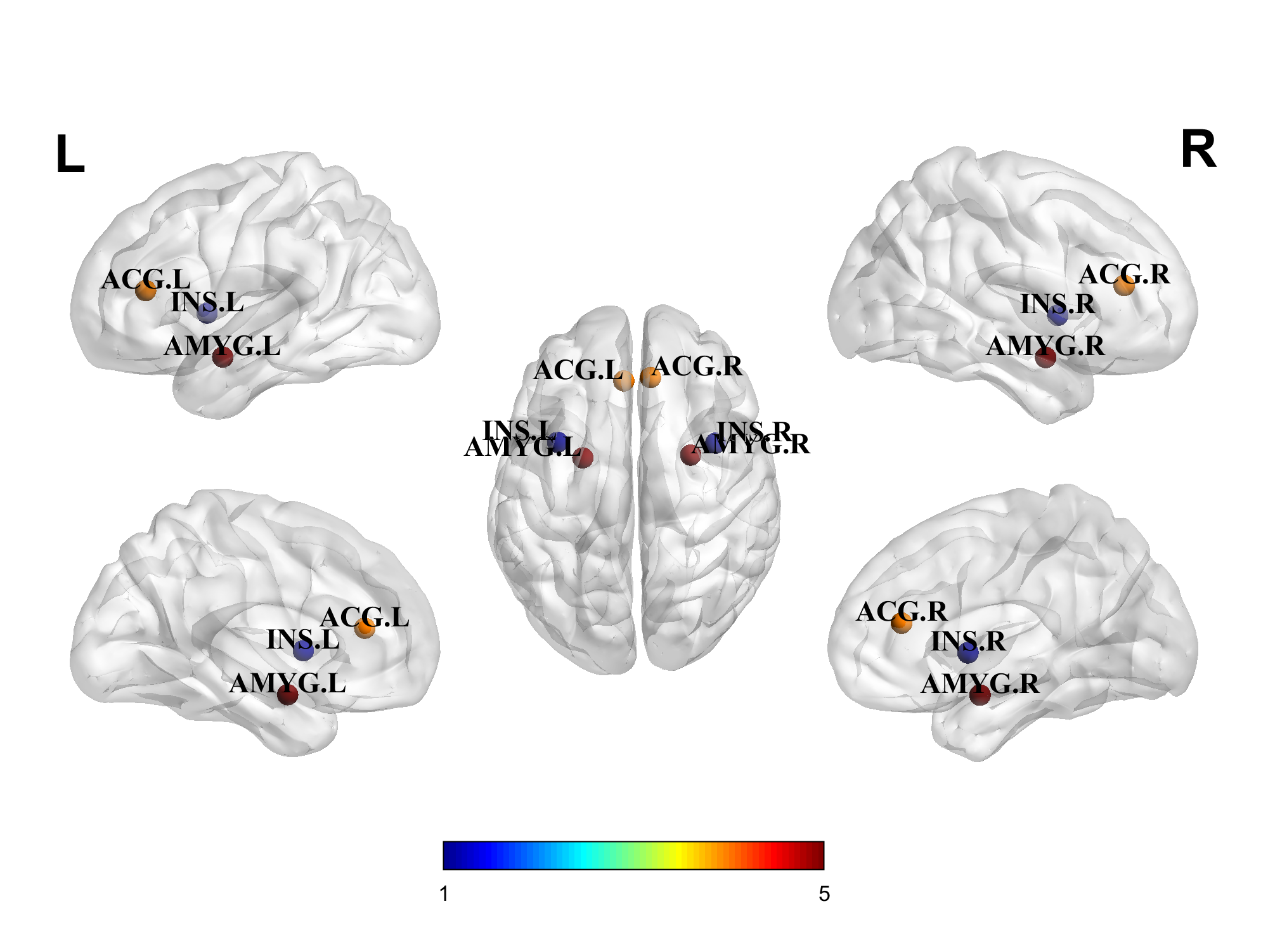


Example of ROI. Amygdala (AMYG), Insula (INS), Anterior cingulate and paracingulate gyri (ACG)

**REFERENCES**

1. Carretié L, Hinojosa JA, Albert J, López-Martín S, De La Gándara BS, Igoa JM, et al. Modulation of Ongoing Cognitive Processes by Emotionally Intense Words. *Psychophysiology* (2008) 45(2):188-96. Epub 2007/11/01. doi: 10.1111/j.1469-8986.2007.00617.x.

2. Moll J, de Oliveira-Souza R, Moll FT, Ignacio FA, Bramati IE, Caparelli-Daquer EM, et al. The Moral Affiliations of Disgust: A Functional Mri Study. *Cogn Behav Neurol* (2005) 18(1):68-78. Epub 2005/03/12. doi: 10.1097/01.wnn.0000152236.46475.a7.

3. Luo Y, Shen W, Zhang Y, Feng TY, Huang H, Li H. Core Disgust and Moral Disgust Are Related to Distinct Spatiotemporal Patterns of Neural Processing: An Event-Related Potential Study. *Biological psychology* (2013) 94(2):242-8. Epub 2013/07/03. doi: 10.1016/j.biopsycho.2013.06.005.

4. Scott GG, O'Donnell PJ, Leuthold H, Sereno SC. Early Emotion Word Processing: Evidence from Event-Related Potentials. *Biological psychology* (2009) 80(1):95-104. Epub 2008/04/29. doi: 10.1016/j.biopsycho.2008.03.010.

5. Beck AT, Steer RA, Ball R, Ranieri WF. Comparison of Beck Depression Inventories-Ia and-Ii in Psychiatric Outpatients. *J Pers Assess* (1996) 67(3):588-97. doi: 10.1207/s15327752jpa6703_13.

6. Du Q, Liu H, Yang C, Chen X, Zhang X. The Development of a Short Chinese Version of the State-Trait Anxiety Inventory. *Front Psychiatry* (2022) 13:854547. Epub 2022/05/28. doi: 10.3389/fpsyt.2022.854547.

7. Eaves L, Eysenck H. The Nature of Extraversion: A Genetical Analysis. *J Pers Soc Psychol* (1975) 32:102-12. doi: 10.1037/h0076862.

8. Hidalgo-Lopez E, Zeidman P, Harris T, Razi A, Pletzer B. Spectral Dynamic Causal Modelling in Healthy Women Reveals Brain Connectivity Changes Along the Menstrual Cycle. *Commun Biol* (2021) 4(1):954. Epub 2021/08/12. doi: 10.1038/s42003-021-02447-w.

9. Seeley WW, Menon V, Schatzberg AF, Keller J, Glover GH, Kenna H, et al. Dissociable Intrinsic Connectivity Networks for Salience Processing and Executive Control. *The Journal of neuroscience : the official journal of the Society for Neuroscience* (2007) 27(9):2349-56. Epub 2007/03/03. doi: 10.1523/jneurosci.5587-06.2007.

10. Zhou Y, Friston KJ, Zeidman P, Chen J, Li S, Razi A. The Hierarchical Organization of the Default, Dorsal Attention and Salience Networks in Adolescents and Young Adults. *Cerebral cortex (New York, NY : 1991)* (2018) 28(2):726-37. Epub 2017/11/22. doi: 10.1093/cercor/bhx307.

11. Engman J, Sundström Poromaa I, Moby L, Wikström J, Fredrikson M, Gingnell M. Hormonal Cycle and Contraceptive Effects on Amygdala and Salience Resting-State Networks in Women with Previous Affective Side Effects on the Pill. *Neuropsychopharmacology* (2018) 43(3):555-63. doi: 10.1038/npp.2017.157.

12. Whitfield-Gabrieli S, Nieto-Castanon A. Conn: A Functional Connectivity Toolbox for Correlated and Anticorrelated Brain Networks. *Brain connectivity* (2012) 2(3):125-41. Epub 2012/05/31. doi: 10.1089/brain.2012.0073.

13. Xia, M., Wang, J., & He, Y. (2013). BrainNet Viewer: A Network Visualization Tool for Human Brain Connectomics. PLOS ONE, 8(7), e68910. doi:10.1371/journal.pone.0068910
